# The Involvement of Ser1898 of the Human L-Type Calcium Channel in Evoked Secretion

**DOI:** 10.1155/2011/746482

**Published:** 2011-12-20

**Authors:** Niv Bachnoff, Moshe Cohen-Kutner, Daphne Atlas

**Affiliations:** Department of Biological Chemistry, The Alexander Silberman Institute of Life Sciences, The Hebrew University of Jerusalem, Jerusalem 919104, Israel

## Abstract

A PKA consensus phosphorylation site S1928 at the **α**_1_1.2 subunit of the rabbit cardiac L-type channel, Ca_V_1.2, is involved in the regulation of Ca_V_1.2 kinetics and affects catecholamine secretion. This mutation does not alter basal Ca_V_1.2 current properties or regulation of Ca_V_1.2 current by PKA and the beta-adrenergic receptor, but abolishes Ca_V_1.2 phosphorylation by PKA. Here, we test the contribution of the corresponding PKA phosphorylation site of the human **α**_1_1.2 subunit S1898, to the regulation of catecholamine secretion in bovine chromaffin cells. Chromaffin cells were infected with a Semliki-Forest viral vector containing either the human wt or a mutated S1898A **α**_1_1.2 subunit. Both subunits harbor a T1036Y mutation conferring nifedipine insensitivity. Secretion evoked by depolarization in the presence of nifedipine was monitored by amperometry. Depolarization-triggered secretion in cells infected with either the wt **α**_1_1.2 or **α**_1_1.2/S1898A mutated subunit was elevated to a similar extent by forskolin. Forskolin, known to directly activate adenylyl-cyclase, increased the rate of secretion in a manner that is largely independent of the presence of S1898. Our results are consistent with the involvement of additional PKA regulatory site(s) at the C-tail of **α**_1_1.2, the pore forming subunit of Ca_V_1.2.

## 1. Introduction

The voltage-gated calcium channels (VGCCs) are regulated by kinases that are activated by second messenger pathways [1–4]. The dihydropyridine-sensitive VGCC, known as the L-type channel or Ca_V_1.2, is modulated by protein kinase A (PKA) and protein kinase C (PKC) [4]. The *β*-adrenergic receptor activates cAMP-dependent phosphorylation by PKA, which increases the amplitude of voltage-dependent Ca^2+^ currents. It is now well established that the *β*-adrenergic signaling enhances inward Ca^2+^ currents via Ca_V_1.2 phosphorylation in cardiac myocytes [2, 4–6], adrenal chromaffin cells [7, 8], rat hippocampal neurons [9] and in skeletal muscle [10].

PKA phosphorylation of the L-type channel leads to an increase in the open probability (*P*_*o*_) of the channel [5] and an increase in the mean open time by augmenting the number of long openings [11]. In the heart activation of the *β*-adrenergic signaling pathways increases calcium currents ~4-fold and is attributed to the phosphorylation of Ca_V_1.2 [12].

The L-type channel assembles with Gs, adenylyl cyclase, PKA, and phosphatase PP2A into a signaling complex that enables the rapid modulation of the channel by the adrenergic receptor [13]. Biochemical studies in the heart have shown that the distal C-terminal tail of the *α*_1_1.2 subunit is phosphorylated by PKA on serine 1928 (S1928) [14–17]. Phosphorylation of S1928 in cardiac and neuronal cells implies *β*-adrenergic regulation of Ca_V_1.2 [4, 17–21].

The phosphorylation of S1928, the PKA consensus phosphorylation site at the *α*_1_1.2 pore forming subunit of rabbit Ca_V_1.2 that is phosphorylated both *in vitro* and *in vivo*, was suggested to be responsible for the increase in Ca^2+^ currents [19].

Additional phosphorylation sites that can also participate in the *β*-adrenergic regulation of cardiac L-type Ca^2+^ channels [16] include the intracellular *β*-subunit of the channel [22, 23]. Since S1928 is phosphorylated also by PKC, it was suggested that both PKA and PKC signaling pathways converge at S1928 of the *α*_1_1.2 subunit to increase channel activity [24].

More recently, the regulation of Ca_V_1.2 by the *β*-adrenergic pathway in mouse heart was tested *in vivo* using knock-in mouse with a targeted mutation of S1928 to alanine [25]. This mutation, which abolished PKA phosphorylation of S1928, did not affect the kinetic characteristics and the *β*-adrenergic regulation of the L-type current. The conclusion led to the suggestion that PKA phosphorylation of S1928 in cardiomyocytes is functionally not involved in the *β*-adrenergic stimulation of Ca_V_1.2-mediated calcium influx [25].

In neuroendocrine cells such as bovine chromaffin and PC12 cells, membrane depolarization triggers catecholamine (CA) secretion primarily by the opening of the L-type channels [26–28]. In these cells, the channel is an integral part of the signaling complex that triggers secretion [29–32]. Voltage-driven perturbations of a Ca^2+^-bound channel were suggested to transmit the signal from the channel to the exocytotic machinery and trigger fast release of vesicles assembled with the channel [28, 31, 33–35]. Therefore, channel phosphorylation would be expected to modify secretion. As previously shown, inhibitors of protein phosphorylation or injection of phosphatase 2A into cells suppresses facilitation of depolarization-evoked secretion [7]. Based on these results it was suggested that facilitation is mediated by phosphorylation of voltage-dependent L-type Ca^2+^ channel [8].

We examined the modulation of L-type channel and its role in mediating evoked secretion using forskolin. Secretion was triggered in cells infected with the human wt *α*_1_1.2 (Hu/wt) or *α*_1_1.2/S1898A (Hu/S1898A) mutant subunit. The S1898 site corresponds to S1928 of rabbit cardiac cells. The frequency of the secretory events mediated by Hu/S1898A and Hu/wt-infected cells was accelerated by forskolin to a similar extent, indicating that mutating serine 1898 to alanine did not affect the forskolin-induced increase of catecholamine secretion.

Overall, our results suggest that additional PKA-sensitive phosphorylation sites contribute to the PKA modulation of catecholamine release.

## 2. Experimental Procedures

### 2.1. Materials and Methods

The complete human cDNAs of *α*_1_1.2 subunit, accession number AJ224873, rabbit *β*2b accession number X64298, and rabbit *α*2/*δ* accession number NM_001082276, were kindly donated by Dr. M. Sanguinetti (University of Utah). A modified pSFV1 (Invitrogen) plasmid, where an internal ribosome entry site from poliovirus had been inserted followed by the gene for enhanced GFP, was provided by Dr. U. Ashery.

**Mutagenesis**

Standard methods of plasmid DNA preparation were used to prepare the human *α*_1_1.2/S1989A. The Ca_V_1.2*α*_1 _subunit coding sequence (AJ224873) was used as a template. The S1898A mutation was introduced by mutating the codon TCC to GCC by using the Bio-X-Act Long DNA Polymerase kit (Bioline, Israel) and Quick-change site-directed mutagenesis PCR was applied (Bioline, Israel). The following primers were used: 5′-TGCCTCACTAGGTCGAAGGGCCGCCTTCCACCTGGAATGTCTGAA and the 3′-TTCAGACATTCCAGGTGGAAGGCGGCCCTTCGACCTAGTGAGGCA. For amperometry studies, plasmids *α*_1_1.2/T1036Y and *α*_1_1.2/S1898/T1036Y were inserted upstream of the internal ribosome entry site using BamHI and BssHII restriction sites. All constructs were verified by DNA sequencing and restriction site mapping.

#### 2.1.1. Chromaffin Cell Preparation and Culture

Bovine adrenal glands were obtained at a local slaughterhouse. The adrenal medulla cells were isolated as previously described in [34] and plated at a density of 5 × 10^4^ cells/cm^2^on glass cover slips placed in 35 mm plates, in DMEM (Gibco) supplemented with ITS-X (Sigma, Israel). Cells were incubated at 37°C in 5% CO_2_  and were used for amperometric recordings 1–4 days after preparation at 23°C.

#### 2.1.2. Amperometric Recordings of Catecholamine Release from Chromaffin Cell

Amperometry recordings were carried out using 5 *μ*m thin carbon fiber electrodes (CFE ALA Incorp. Westbury, NY, USA) and a VA-10 amplifier (NPI-electronic, Tamm, Germany) held at 800 mV as described previously [36]. Cells were rinsed 4 times prior to the experiment and bathed during the recordings at 23°C in isoosmotic physiological solution (149 mM NaCl, 2 mM KCl, 1 mM MgCl_2_, 2 mM CaCl_2_, 10 mM Glucose, and 10 mM HEPES, pH 7.3 at ~23°C (adjusted with NaOH)). Individual cells were stimulated to release by a 10 sec application of isoosmotic 60 mM KCl buffer from a ~3 *μ*m tipped micropipette placed 5–7 *μ*m from the cell in the bath. Amperometric currents were sampled at 10 kHz, using clampex 9.2 (Axon Instruments) and low-pass filter at 1 kHz.

Secretion of catecholamines from fluorescent cells identified at 480 nm excitation was recorded 16–24 hrs after infection by amperometry.

#### 2.1.3. pSFV Infection

**Virus Preparation and Infection**

Recombinant SFV particles were generated as described previously in [37]. Briefly, *in vitro* transcribed RNA from pSFV-GFP, or pSFV expressing GFP through an internal ribosome entry site (IRES) motif, pSFV-Nifedipine-insensitive *α*_1_1.2-IRES-GFP, or pSFV-Nifedipine-insensitive *α*_1_1.2/S1898A-IRES-GFP was coelectroporated into BHK-21 cells with pSFV-Helper-2 RNA. Virus stocks were harvested 24 hr later and activated with alpha-chymotrypsin before infection studies. Approximate titers were estimated by infection of known numbers of BHK-21 cells with serial dilutions of SFV stocks, and the GFP-positive cells were counted. Generally, titers in the range of 5 × 10^8^ infectious particles/mL were obtained.

SFV particles (30 *μ*L/dish) were added to the cultured cells (2 × 10^5^ cells per dish) 5–48 h after plating. Infected cells were identified by their GFP fluorescence.

#### 2.1.4. Amperometric Data Acquisition and Analysis

Rates of secretion were determined for individual cells and averaged. Spike frequencies were quantified for individual cells by the slopes of the cumulative events during 10 sec of KCl stimulation and a 20 sec poststimulus period (10–40 sec) and were averaged to obtain a cell mean. The rate of secretion during 10–40 sec was determined from the slopes of the corresponding cumulative spike plots. Data was analyzed as described in the text and figure legends. Error bars give standard errors. Spikes exceeding three times the background noise (>10 pA) were analyzed. All peaks identified by the program IGOR PRO (Wavemetrics, Lake Oswego, Or, USA) were inspected visually and bad signals were excluded manually.

#### 2.1.5. Expression in Xenopus Oocytes and cRNA Injection

Stage V-VI *Xenopus laevis* oocytes were removed surgically from the ovaries of anesthetized animals and transferred to a Ca^2+^-free medium (96 mM NaCl, 2 mM KCl, 1 mM MgCl_2_, and 5 mM HEPES, pH7.4) containing 1 mg/mL collagenase (253 U/mg) (Wortington Bioche. Corp. USA). The follicular cell layer was removed by shaking the oocytes in this buffer for 1.5–2 hr at room temperature. After extensive wash, the oocytes were transferred to ND96 buffer (96 mM NaCl, 3 mM KCl, 1 mM MgCl_2_, 1.8 mM CaCl_2_, and 5 mM HEPES, pH7.4) containing 2.5 mM pyruvate, 100 U/mL penicillin, and 10 *μ*g/mL streptomycin. Oocytes were incubated in ND96 for 12–20 hr before cRNA injection.

#### 2.1.6. cRNA Injection into Oocytes and Electrophysiology

cRNAs were prepared using the T7 Fermentas transcription kit (Lituania), and the product was monitored by gel electrophoresis and optical density measurements. *In vitro* transcribed capped cRNA of the channel subunits were injected into the defolliculated oocytes in a final volume of 40 nL using a Drummond 510 microinjector (Broomall, Pa, USA). Oocytes were maintained at 18°C for 5 days after injection [30].

#### 2.1.7. Confocal Imaging

Single optical sections through the oocytes were obtained with an Olympus FV1000 (Olympus, Japan) equipped with a 40x oil objective (N.A. 1.3). Two excitation lasers were used sequentially: 488 nm GFP and narrow-band emission filters 505–525 nm. Sequential scanning was performed with a resolution set to 512 × 512 pixels (0.621 mm/pixel), and single optical sections ~0.5 *μ*m thick were captured. Exposure time was 8 *μ*sec/pixel.

## 3. Results

### 3.1. Mutated *α*_1_ Subunit Is Targeted to the Cell Membrane

**Figure 1:**
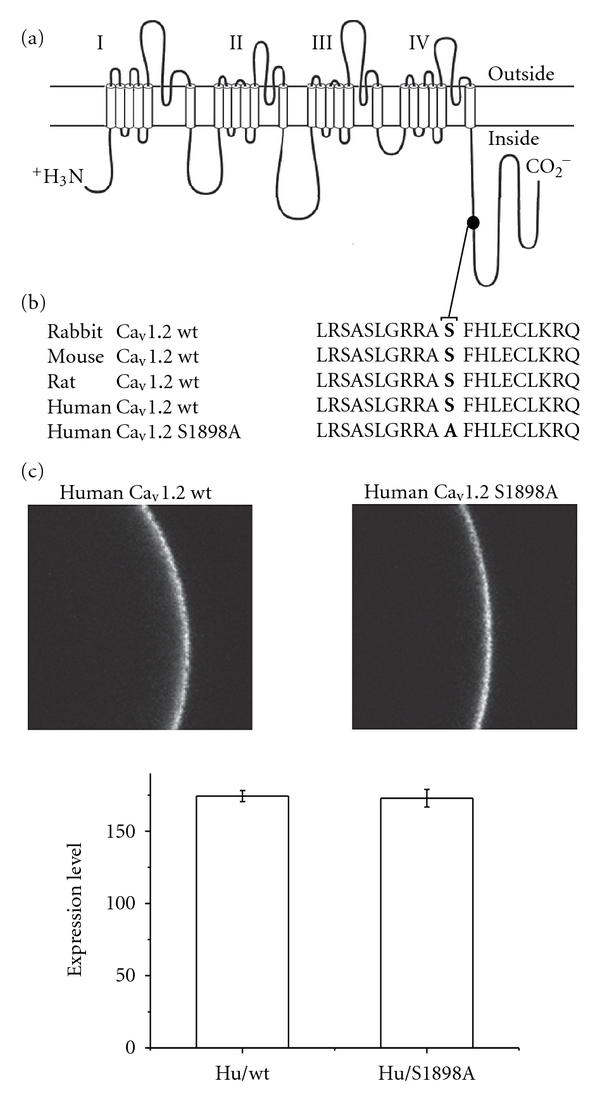
Membrane targeting of *α*_1_1.2 and *α*_1_1.2/S1898A to the membrane. (a) Schematic view of the *α*_1_1.2 pore forming subunit of Ca_V_1.2. (b) A comparison of the protein kinase A (PKA) consensus sequence of the highly conserved Ser located at the distal C-terminal of *α*_1_1.2 of various species. (c) The human GFP-*α*_1_1.2 (7 ng/oocyte) and the GFP-*α*_1_1.2/S1898A (7 ng/oocyte) were injected into *Xenopus laevis* oocytes together with the *β*2b (2.5 ng/oocyte) and *α*2*δ* (5 ng/oocyte). Five days later the oocytes were imaged by confocal microscopy. Membrane targeting and expression of the channels to the membrane were determined by calculating the pixel/area.

The sequence homology of the phosphorylation consensus site of S1928 at the distal C-terminal of the *α*_1_1.2 pore forming subunit corresponds to S1898 in the human *α*_1_1.2, as shown in Figure 1(a). This highly conserved sequence in various species represents a PKA consensus phosphorylation motif (Figure 1(b)).

The consensus S1898 of the human *α*_1_1.2 subunit was mutated to Ala and inserted into a frog expression vector. The cRNAs encoding the GFP-tagged human *α*_1_1.2 subunit of Ca_V_1.2 or the GFP-*α*_1_1.2/S1898A mutant were coinjected into oocytes along with the cRNAs encoding the auxiliary rabbit *α*2*δ* and *β*2b channel subunits (see Section 2). Oocytes imaged by confocal microscopy showed a similar mean gray value for both channels, indicating nearly identical level of protein expression and targeting to the membrane (Figure 1(c)).

### 3.2. Depolarization-Induced Secretion in Bovine Chromaffin Cells Is Largely Mediated by Ca_V_1.2 and Is Not Affected by the Viral Infection

Modulation of catecholamine secretion mediated by human *α*_1_1.2 subunit was tested in bovine chromaffin cells. 

**Figure 2:**
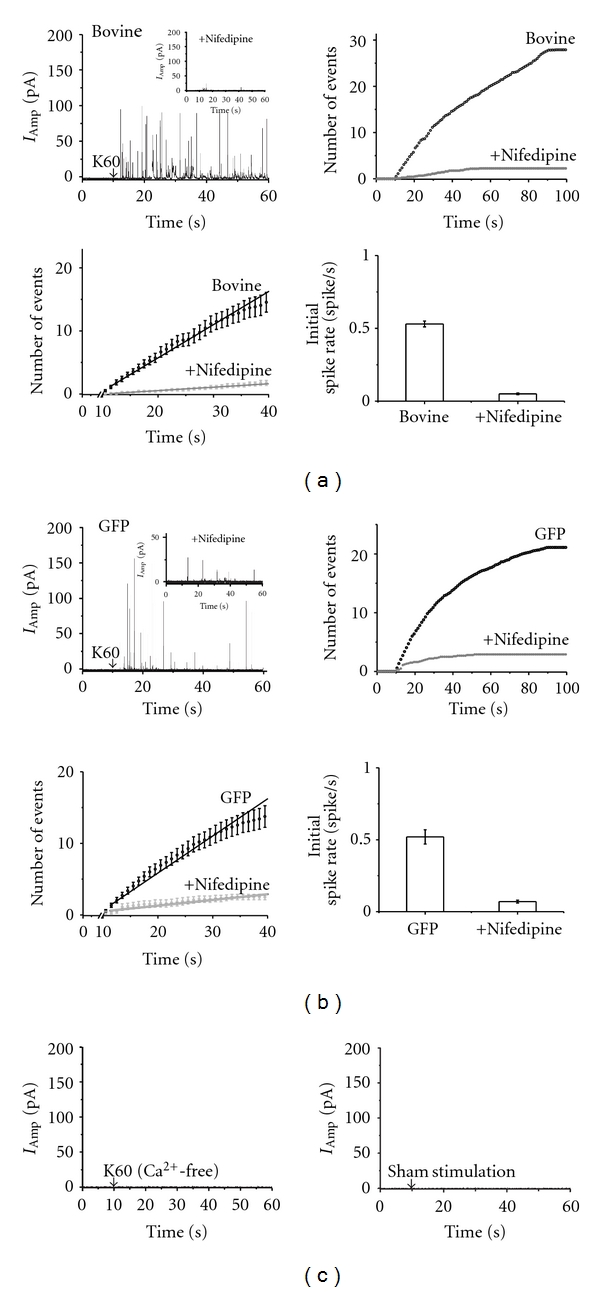
Amperometry measurements of catecholamine release in bovine chromaffin cells. (a) Traces of amperometry currents elicited in noninfected chromaffin cells by a 10 sec puff of 60 mM KCl (K60) as indicated by the arrow (*left*), and in the presence of 5 *μ*M nifedipine (*inset*). The cumulative number of events per cell (*n* = 18), averaged with and without 5 *μ*M nifedipine, was plotted *versus* time (*right). *An expanded view of the initial cumulative spike counts (*lower, left*) and the initial rate of secretion calculated from the linearization of the cumulative averaged spike count (0.53 ± 0.02 spike/sec; *lower*, *right*). (b) Traces of amperometry currents elicited by a 10 sec puff of 60 mM KCl (K60) in cells infected with pSFV containing GFP (*left*) and in the presence of 5 *μ*M nifedipine (*inset*). The cumulative number of events per cell was averaged (*n* = 49) with and without 5 *μ*M nifedipine and plotted *versus* time (*right*). An expanded view of the initial cumulative spike counts (*lower, left*) and the initial rate of secretion calculated from the linearization of the cumulative averaged spike count (0.52 ± 0.01 spike/sec; *lower, right*). (c) Bovine chromaffin cells stimulated under the same experimental conditions as above in nominally Ca^2+^ free solution (*left*) or stimulated with isoosmotic solution containing 2 mM KCl a sham stimulation (*right*).

At first, using a selective Ca_V_1 channel blocker, we confirmed that depolarization-evoked release is mediated primarily by the L-type channel (Figure 2(a)). The evoked release was significantly inhibited by 5 *μ*m nifedipine present in the bath (Figure 2(a) *inset*). Averaged time courses of exocytosis in the absence and in the presence of 5 *μ*M nifedipine are shown by the cumulative histograms (Figure 2(a), *upper right*). Spike frequencies were quantified for individual cells as initial rates, which correspond to the linear fits to the averaged cumulative histograms (bold lines) collected from cells (*n* = 49) during the 10 sec of 60 mM KCl (K60) stimulation and a 20 sec poststimulus period (range 10–40 sec) (Figure 2(a), *lower right*).

We then tested the effect of Semliki Forest virus (SFV) infection on the rate of secretion in cells using SFV viral vector harboring GFP, as previously described [28]. The infected fluorescent cells that were visualized using UV detection were depolarized by K60 for 10 sec and catecholamine release was monitored as amperometric spikes using carbon fiber electrode, where each spike represents the release of a single vesicle [36, 38]. Secretion from the fluorescent cells was monitored 16–24 hrs after infection. The extent of release was hardly affected by the infection procedure as seen in the amperometric traces (Figure 2(b)) consistent with previous studies [28, 37]. Evoked secretion was significantly decreased in the presence of nifedipine, in GFP-infected cells, similarly to noninfected cells (Figure 2(b) *inset*). The overall time course of secretion determined from the normalized waiting time distributions was 0.52 spike/sec in the GFP-infected cells (Figure 2(b), *lower*) and 0.53 spike/sec in noninfected cells (Figure 2(a), *lower*). No release was detected when the cells were stimulated in a Ca^2+^-free solution (Figure 2(c), *left*) or in the absence of K60 (Figure 2(c), *right*).

### 3.3. The Kinetic Parameters of Amperometric Spikes and Foot in Cells Infected with GFP- and the wt Human *α*_1_1.2 (Hu/wt)

**Figure 3:**
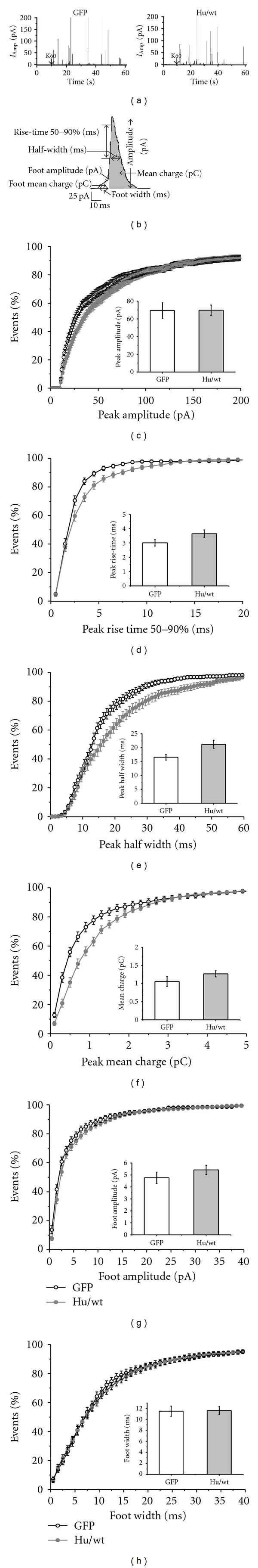
The kinetics of spike and foot parameters elicited in GFP- and human-wt *α*_1_1.2-(Hu/wt-) infected cells are similar. (a) Amperometric currents representing single-vesicle events were triggered by a 10 sec pulse of K60 in cells infected with pSFV containing GFP or Hu/wt in the presence of 5 *μ*M nifedipine. (b) The kinetic properties of a single amperometric event: peak amplitude, half-width, 50–90% rise time, and integrated spike (*Q, grayarea*). Foot signal: foot-width, foot-amplitude, and integrated foot (*Q, whitearea*). (c) Cumulative distribution plots of spike number and analysis of peak amplitude induced in GFP- or Hu/wt-infected cell; (*inset*), mean values for peak amplitude *n* = 1048, *n* = 943 events per data point, respectively. (d) Cumulative distribution plot of spike number and analysis of 50–90% rise time detected in GFP- and Hu/wt-infected cells. Data plotted in a similar fashion as in (c); mean values for 50–90% rise time (*inset*). (e) Cumulative distribution plot of spike number and analysis of half-width in GFP- or Hu/wt-infected cells (*inset*), mean values for spike half-width (f) Cumulative distribution plot of spike number and analysis of mean charge in GFP- or Hu/wt infected cells; (*inset*), mean values for spike mean charge. (g) Cumulative distribution plot of foot number and analysis of foot amplitude in GFP- or Hu/wt-infected cells (*inset*), mean values for foot amplitude. (h) Cumulative distribution plot of foot number and analysis of foot width in GFP- or Hu/wt-infected cells (*inset),* mean values for foot width *P < 0.05.

To study secretion mediated by the human channel, we created a pSFV vector expressing wt *α*_1_1.2 human channel subunit (Hu/wt). As a control we monitored the kinetics of secretion induced in cells that were infected with pSFV vector expressing GFP (see above). Secretion mediated by the wt *α*_1_1.2(Hu/wt) pSFV vector was monitored in the presence of 5 *μ*M nifedipine. The nifedipine insensitivity was used to distinguish between the endogenous cellular channels and our cloned channels that were made resistant to nifedipine (Nif) by a single-amino-acid mutation, T1036Y [39]. The T1036Y mutation rendered the channels completely impermeable to calcium ions as previously shown in HEK 293 cells and in *Xenopus* oocytes [28, 39]. Differences between the spike and pre-spikes (foot) kinetics elicited in GFP-infected and the Hu/wt-infected cells indicate differences between bovine and human Ca_V_1.2. Representative amperometric spikes elicited by a 10 sec pulse of K60 from single GFP- and in Hu/wt-infected cells are shown in Figure 3(a). The kinetic parameters of amperometric currents, including peak amplitude, half-width, 50–90% rise time, and integrated spike (*Q*, *gray area*) as well as foot width, foot amplitude, and integrated foot (*Q*, *white area*), are presented schematically in Figure 3(b). The amperometric currents were quantified analyzing the distribution of the data depicted as cumulative probability in the GFP and the Hu/wt channel subunit (Figures 3(c)–3(f)). For every parameter, the values from each cell were averaged and presented as the mean of cell averages ± the standard error of the mean (SEM) for each group [40]. The kinetic parameters of spikes including spike amplitude, 50–90% rise time, and mean-charge, were similar while the spike half-width was slightly higher in Hu/wt (Figures 3(e) and 3(f)).

The foot amplitude and the kinetics of foot width were not significantly different, calculated by analyzing the distribution of the data and depicted as cumulative probability in the GFP- and the Hu/wt-infected cells (Figures 3(g) and 3(h)).

### 3.4. The Kinetic Parameters of Amperometric Spike and Foot Elicited by Human wt and Mutated S1898A Channels

**Figure 4:**
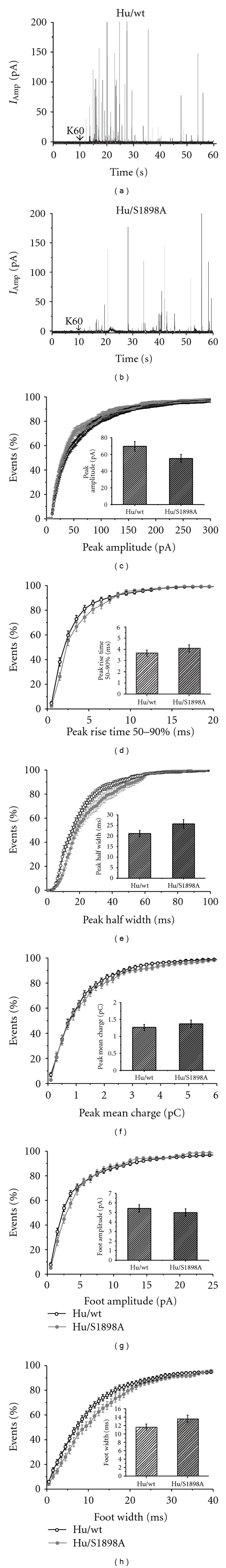
Spike and foot parameters of Hu/wt are not affected by the S1898A mutation. Amperometric current triggered in the presence of 5 *μ*M nifedipine by a 10 sec pulse of K60 as indicated by the arrow. (a) Representative single amperometric events triggered in cells infected with Hu/wt subunit (*n* = 43) or (b) Hu/S1898A (*n* = 42). (c) Cumulative distribution plot of spike number and analysis of peak amplitude induced in Hu/wt- and Hu/S1898A-infected cells (*inset)* mean values for peak amplitude *n* = 1048, *n* = 547 events per data point, respectively. (d) Cumulative distribution plot of spike number and analysis of 50–90% rise time detected in Hu/wt- and Hu/S1898A-infected cells. Data plotted similar fashion as in panel (c), mean values for 50–90% rise time (*inset*). (e) Cumulative distribution plot of spike number and analysis of half-width of Hu/wt- and Hu/S1898A-infected cells; mean values for spike half-width (*inset*). (f) Cumulative distribution plot of spike number and analysis of mean charge in Hu/wt- and Hu/S1898A-infected cells; mean values for spike mean charge (*inset*). (g) Cumulative distribution plot of foot number and analysis of foot amplitudes in Hu/wt- and Hu/S1898A-infected cells; mean values for foot amplitude (*inset*). (h) Cumulative distribution plot of foot number and analysis of foot width in Hu/wt- and Hu/S1898A-infected cells; mean values for foot width  (*inset*).

The *α*_1_1.2 (Hu/wt) or the *α*_1_1.2/S1898A (Hu/S18989A) subunits that harbored a second mutation T1036Y that rendered the channel nifedipine insensitive were subcloned into a pSFV, which expresses GFP through an internal ribosome entry site (IRES) motif [28]. Both Hu/wt and the mutated Hu/S1898A subunits, which included GFP, were made dihydropyridine insensitive and enabled discrimination of secretion mediated by exogenously expressed human channels from that of native bovine calcium channels [28, 39]. We tested the kinetic parameters of cells infected with either Hu/wt or Hu/S1898A subunits in the presence of 5 *μ*M nifedipine, 16–24 hrs after infection as shown by representative amperometric currents (Figures 4(a) and 4(b)). The amperometric spikes elicited in the Hu/wt, and Hu/S1898A-infected cells were quantified by analyzing the distribution of the data, depicted as cumulative probability (Figures 4(c)–4(f); see also Figure 3). For every parameter, the values from each cell were averaged and presented as the mean of cell averages ± the standard error of the mean (SEM) for each group [40]. The mutation of Ser 1898 to Ala, which prevents the phosphorylation at this site, enabled the examination of the net effect of this site on the kinetics of spike and foot parameters. The mutation did not affect spike and foot kinetics as shown by the mean distribution of peak amplitude, half-width, charge, or 50−90% rise-time (Figures 4(c)–4(f)).

In addition, foot values that correspond to the pre-spike signal and depict the properties of the fusion pore [41] did not show any significant changes in the mean distribution of the cumulative events (Figures 4(g) and 4(h)).

### 3.5. Forskolin Increases the Rate of Secretion

It has previously been shown that S1928 is phosphorylated by PKA, both *in vitro* and *in vivo, *either directly by *β*-adrenergic agonists or indirectly by forskolin [14, 15].

To gain insight into the specific contribution of the corresponding consensus PKA site S1898 to the regulation of catecholamine release mediated via Ca_V_1.2, we applied forskolin to cells infected with Hu/wt and Hu/S1898A.

**Figure 5:**
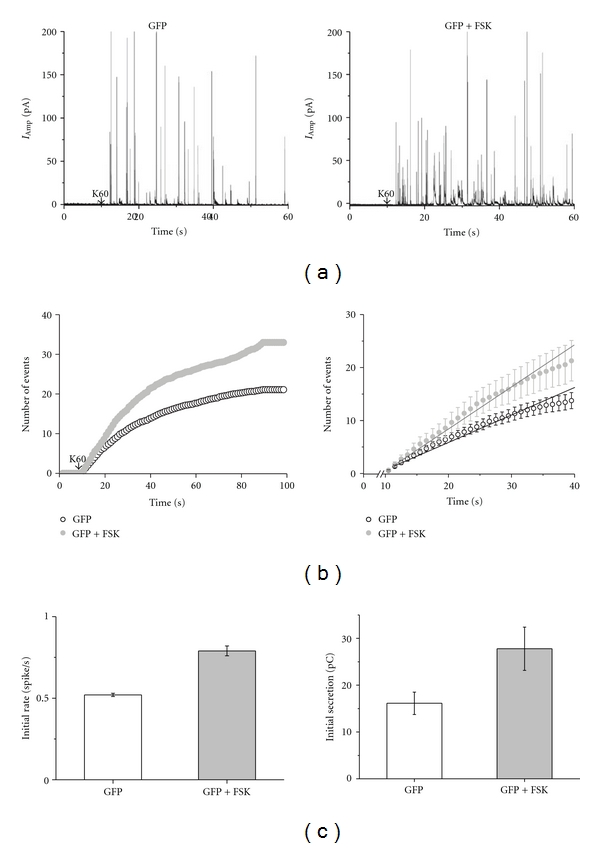
The effect of forskolin on GFP-infected chromaffin cells. Amperometry currents were elicited by a 10 sec puff of 60 mM KCl (K60) in GFP-infected cells in the presence of 1 *μ*M forskolin, as indicated by the arrow. (a) Representative single amperometric events of GFP-infected cells with (*n* = 21) or without forskolin (*n* = 49). (b) The cumulative number of events per cell averaged for GFP-infected cells with or without forskolin was plotted *versus* time (*left*). An expanded view of the initial cumulative spike counts plotted *versus* time (*right*). (c) The initial rate of secretion calculated from the linearization of the cumulative averaged spike count (*left*). Means were calculated for individual cells as an average of more than 500 spikes; see Table 1. The total amount of catecholamine secreted was quantified by averaging of the total mean charge. Summation of spike areas in cells infected with GFP during time range of 10–40 sec, which includes the 10 sec K60 stimulation and a 20 sec poststimulus period (*right*); *P < 0.05.

The effect of forskolin on secretion was tested at first, on GFP-infected cells. As shown by the amperometric currents (Figure 5(a)), 1 *μ*M forskolin when applied to GFP-infected cells increased the frequency of the amperometric events. The analysis of cumulative spikes showed an increase from 21 ± 3 to 33 ± 3.1 spikes/cell (Table 1) and a 1.5-fold increase in the initial rate from 0.52 ± 0.01 to 0.79 ± 0.03 spike/sec (Figure 5(c) *left*). The increase in the frequency of the amperometric events induced by forskolin also led to a 1.6-fold increase in total catecholamine secretion, which was calculated as the total mean charge of spike area within the range of 10–40 sec during and after stimulation (Figure 5(c) *right*; Table 1).

**Figure 6:**
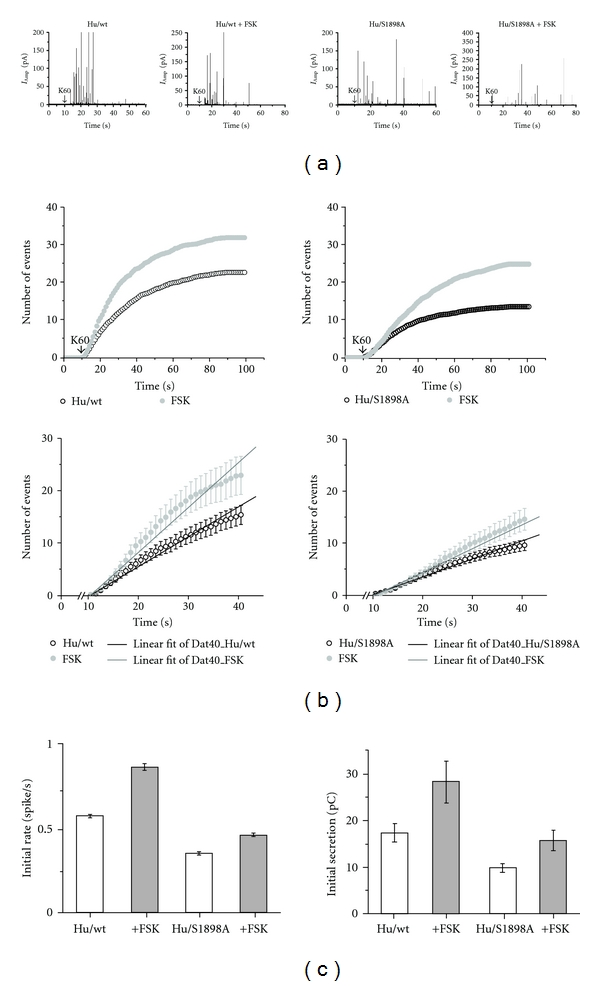
Forskolin accelerates secretion to the same extent in Hu/wt- and Hu/S1898A-infected cells. Amperometry currents were elicited by a 10 sec puff of 60 mM KCl (K60) as indicated by the arrow, in chromaffin cells infected with pSFV containing Hu/wt (*left*) or Hu/S1898A (*right*) in the presence of 1 *μ*M forskolin and 5 *μ*M nifedipine. (a) Representative single amperometric events in Hu/wt or Hu/S1898A infected cells, with and without forskolin. (b) The cumulative number of events per cell averaged for Hu/wt- and Hu/S1898A-infected cells (*n* = 49 and *n* = 42), with or without forskolin (*n* = 43 and *n* = 40), was plotted *versus* time. An expanded view of the initial cumulative spike counts plotted *versus* time (*lower*). (c) The initial rates of secretion were calculated from the linearization of the cumulative averaged spike count (*left*). Means were calculated for individual cells as an average from more than 500 spikes; see Table SI. Total amount of catecholamine secreted was quantified by averaging of the total mean charge. Summation of spike areas in cells infected with Hu/wt (empty columns) or Hu/S1898A (gray columns) during time range of 10–40 sec (*right*); *P < 0.05.

Next, we compared the effect of forskolin on secretion mediated by the Hu/wt or Hu/S1898A. Shown by representative amperometric traces, forskolin accelerated the rate of secretion in cells infected with either Hu/wt or Hu/S1898A (Figure 6(a)). The increase in the frequency of the amperometric spikes induced by forskolin was quantified as described above (see Figure 2). In both the GFP- and Hu/wt-infected cells, the rate of secretion was increased 1.5-fold, and in the Hu/S1898A-infected cells 1.3-fold (Figures 6(a) and 6(b) and Table 1). Forskolin increased the total release of catecholamine to a similar extent (1.6-fold), in the GFP-, Hu/wt-, or Hu/S1898A-infected cells, as summarized in the bar graph (Figure 6(c)) and Table 1.

Forskolin induced a similar upregulation of release events mediated by the human Hu/wt and the endogenous bovine channel (measured in cells infected with GFP). A smaller increase (1.3-fold compared to 1.5-fold) was observed in Hu/S1898A-infected cells.

### 3.6. The Effects of Forskolin on the Kinetics of the Amperometric Spikes and Foot

In spite of the increase in the frequency of secretion by forskolin, no effect on the kinetics of the individual amperometric spikes and prespikes (foot) was observed, as presented in Table SI of the supplementary material available online at doi:10.1155/2011/746482.

## 4. Discussion

### 4.1. The Contribution of S1898 to the Rate of Secretion in Chromaffin Cells

In bovine chromaffin cells membrane depolarization triggers catecholamine release primarily via the activation of the dihydropyridine-sensitive VGCC, known as the L-type channel. This was previously shown using selective DHP L-type channel blockers [26, 28]. The activity of Ca_V_1.2 like other VGCC is further modulated by phosphorylation [2, 4].

Phosphorylation of the L-type channel or Ca_V_1.2 by protein Kinase A (PKA) and protein kinase C (PKC) further modulates the kinetics of the channel [4]. Biochemical studies in the heart and in neuronal cells have shown that the distal C-terminal tail of the *α*_1_1.2 subunit is phosphorylated by PKA on serine 1928 (S1928) [14–17]. This consensus phosphorylation site corresponds to S1898 in the human Ca_V_1.2, and here we have tested whether this site at the human pore forming *α*_1_1.2 subunit contributes to the regulation of catecholamine release in bovine chromaffin cells.

At first we confirmed that bovine chromaffin cells infected with pSFV vector containing GFP retained their sensitivity to nifedipine. Secretion of CA that was monitored using carbon fiber amperometry was reduced by more than 85%.

Both the human wt *α*_1_1.2 (Hu/wt) and the *α*_1_1.2/S1898A (Hu/S1898A) subunits, which are equally expressed and targeted to the cell membrane, were introduced into chromaffin cells via Semliki forest viral infection. The two channel subunits each harbored an additional mutation that rendered them nifedipine insensitive. Secretion in the infected cells was carried out in the presence of nifedipine, which enabled the functional discrimination of L-type-mediated secretion contributed by exogenously expressed human channels from that of native bovine Ca^2+^ channels. The frequency of secretion and the total catecholamine released that was mediated by the endogenous bovine channel was similar to that of the human *α*_1_1.2 (Hu/wt) channel. Also the spike parameters and the prespike (foot) parameters were indistinguishable.

### 4.2. Forskolin Elevated Depolarization-Evoked Secretion Independently of S1898

Protein microsequencing of phospho-peptide mapping confirmed that phosphorylation occurs at a PKA site, at serine 1928 near the C-terminus of cardiac *α*_1_1.2 [14]. Based on these results it was suggested that the single PKA consensus site S1928 is involved in the *β*-adrenergic receptor modulation of channel activity.

Forskolin is known to activate adenylyl cyclase, which by producing cAMP and activating PKA, leads to the phosphorylation of the *α*1 subunits of the L-type channel thereby, upregulating its activity. It was previously shown that forskolin elevation of cAMP could be fully accounted for by the activation of the *β*-adrenergic receptor [25, 42, 43]. Using forskolin, we tested the contribution of the human *α*_1_1.2 subunit to secretion by mutating Ser1898 to Ala, the site that corresponds to the S1928 in rabbit cardiac *α*_1_1.2 subunit.

The modulation of secretion by the PKA activation pathway was tested by forskolin that was applied to chromaffin cells infected with the Hu/wt or Hu/S1898A subunits.

The direct activation of adenylyl-cyclase by forskolin in GFP-infected and in the Hu/wt-infected cells led to a similar fold increase in the rate of depolarization-evoked secretion and total catecholamine release. These results suggest that the human *α*_1_ subunit was most likely phosphorylated by PKA to the same extent as the endogenous *α*_1_ subunit.

On the other hand, the positive impact of forskolin on the rate of secretion was slightly lower in cells infected with the Hu/S1898A subunit. These results suggest that forskolin upregulates evoked secretion only marginally via S1898.

These results are in agreement with others [23] who have demonstrated that the phosphorylation of S1928 in the rabbit channel accounts only partially (~20–30%) for the *β*-adrenergic regulation of calcium currents in cardiomyocytes [17]. Furthermore, in Ca_V_1.2^S1928-129B6F2^ mice, no detectable differences in heart rate were detected compared to control animals during isoproterenol infusion [25]. It is predicted that forskolin mediates phosphorylation through additional site(s) either at the *α*_1_1.2 subunit or in other channel subunits [22, 44] and only marginally through S1898. Previous studies have also shown that phosphorylation of this site involves PKC [24].

In our studies, both the Hu/wt and the Hu/S1898A channel subunits mediate faster rate of secretion by forskolin. Since forskolin modified secretion largely independently of the presence of Ser or Ala at position 1898, we concluded that the cAMP-dependent PKA has contributed to the modulation of secretion via other selective sites [25]. Other studies have proposed that the cardiac L-type Ca^2+^ channels are phosphorylated in basal state by high levels of PKA phosphorylation, while dephosphorylation reduces their activity [19, 45].

In summary, our results show that mutating S1898 hardly affected the forskolin-induced increase in depolarization-evoked secretion in the neuroendocrine cells. Given that a similar forskolin-mediated increase in the rate of secretion was observed in cells infected with Hu/wt and Hu/S1898A mutant, our results indicate that other phosphorylation sites either at the L-type channel auxiliary subunits or other proteins of the secretory apparatus are involved in the upregulation of depolarization-evoked secretion in chromaffin cells.

## Supplementary Material

Supporting material: The kinetic parameters of the spikes and foot of elicited in bovine chromaffin cells infected by pSFV vectors of the **α**_1_11.2 subunits of Hu/wt and Hu/S1898A. The cells were stimulated by a 10 sec puff of 60mM KCl (see Experimental Procedures).

## Figures and Tables

**Table 1:** The effect of forskolin on the rate and total catecholamine secretion. The rate of secretion was determined in cells infected with GFP, Hu/wt, the wt human *α*_1_1.2 subunit, and Hu/S1898A, and the mutant *α*_1_1.2/S1898A subunit.

Group	# Cells	# Spikes	AVG # Spikes	Frequency		Secretion	
				10–40 sec (Spike/sec)	Fold	10–40 sec (pC)	Fold
Noninfected	18	502	27 ± 4.8	0.53 ± 0.02 *R* ^2^ = 0.995		15.4 ± 1.8	

GFP	49	1048	21.4 ± 3.0	0.52 ± 0.01 *R* ^2^ = 0.989		16 ± 2.5	
GFP + FSK^a^	21	693	33 ± 3.2	0.79 ± 0.03 *R* ^2^ = 0.994	1.5	26 ± 4.4*	1.6

Hu/wt	43	943	22 ± 2.5	0.57 ± 0.02 *R* ^2^ = 0.989		17 ± 1.9	
Hu/wt + FSK	34	1052	30 ± 4.9	0.86 ± 0.03 *R* ^2^ = 0.982	1.5	28 ± 4.5*	1.6

Hu/S1898A	42	547	13 ± 1.4	0.35 ± 0.01 *R* ^2^ = 0.995		10 ± 0.9	
Hu/S1898A +FSK	40	953	23.8 ± 3.5	0.46 ± 0.01 *R* ^2^ = 0.985	1.3	16 ± 2.1*	1.6

Cells were stimulated for 10 sec by 60 mM KCl. Amperometric currents were measured during time range of 10–40 sec, which includes the 10 sec K60 stimulation and a 20 sec poststimulus period.

^a^1 *μ*M forskolin (FSK);*P < 0.05.
