# Supplementary figures and images for: The Involvement of Ser1898 of the Human L-Type Calcium Channel in Evoked Secretion
Source: Int J Endocrinol. 2011 Dec 20;2011:746482. doi: 10.1155/2011/746482 (PMC3246732; doi:10.1155/2011/746482)

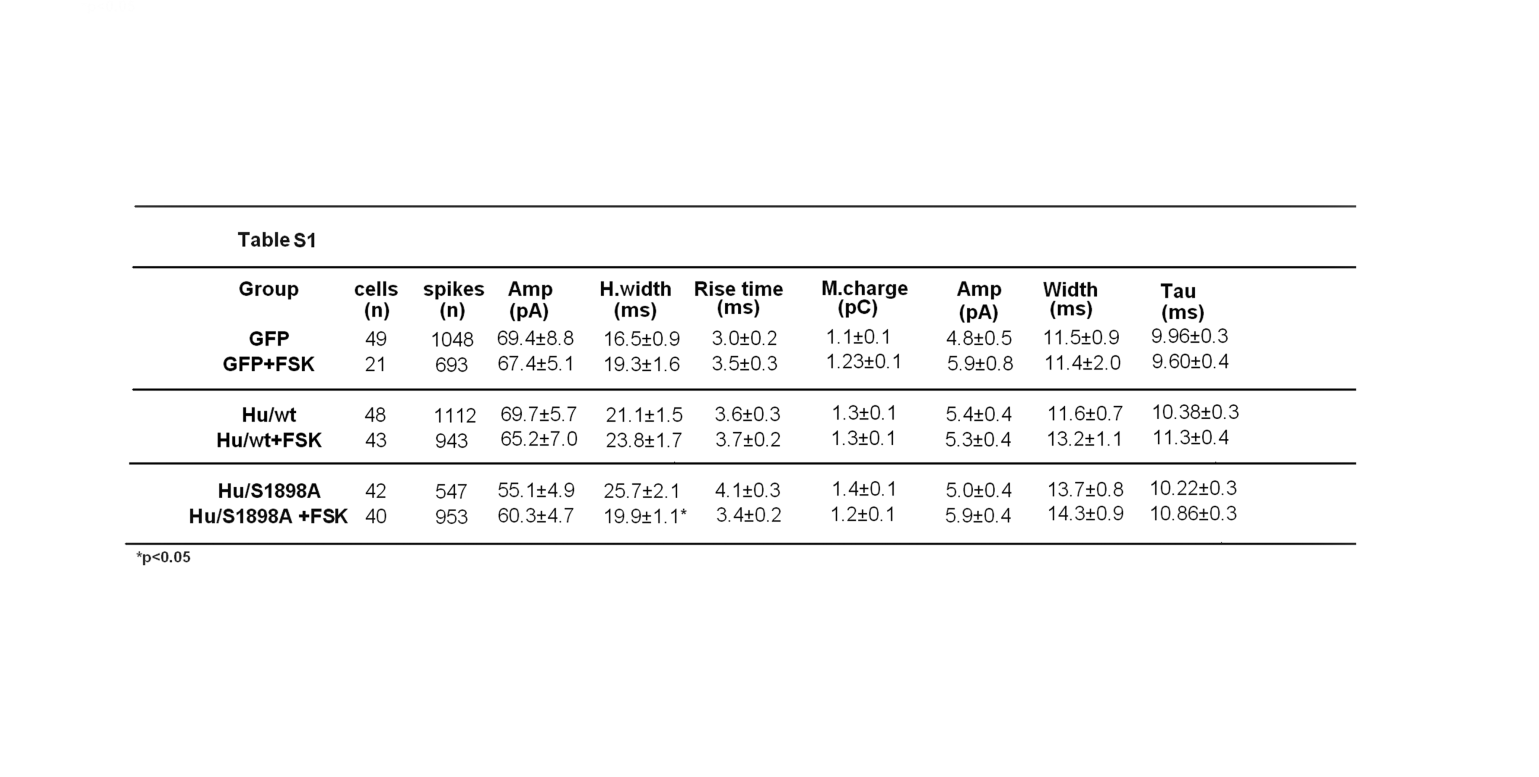

Supplement: Supplementary file 1 — Supporting material: The kinetic parameters of the spikes and foot of elicited in bovine chromaffin cells infected by pSFV vectors of the α 111.2 subunits of Hu/wt and Hu/S1898A. The cells were stimulated by a 10 sec puff of 60mM KCl (see Experimental Procedures). [file 746482.f1.doc]
